# How warm are political interactions? A new measure of affective fractionalization

**DOI:** 10.1371/journal.pone.0294401

**Published:** 2024-05-14

**Authors:** Ansgar Hudde, Will Horne, James Adams, Noam Gidron

**Affiliations:** 1 Institute of Sociology and Social Psychology, University of Cologne, Cologne, Germany; 2 Georgia State University, Atlanta, Georgia; 3 University of California, Davis, Davis, CA, United States of America; 4 The Hebrew University of Jerusalem, Jerusalem, Israel; Lund University: Lunds Universitet, SWEDEN

## Abstract

Affective polarization measures account for partisans’ feelings towards their own party versus its opponent(s), but not for how likely partisans are to encounter co-partisans versus out-partisans. However, the intensity of out-party dislike and the probability with which this comes into play both determine the social impact of cross-party hostility. We develop an affective fractionalization measure that accounts for both factors, and apply it to longitudinal survey data from 20 Western publics. From this perspective, countries with fewer dominant parties may be more harmonious because partisans have lower probabilities of interacting with political opponents. At the party level, partisans of smaller, more radical parties are particularly troubled because they strongly dislike out-partisans and have few co-partisans. Affective fractionalization has increased in most Western publics over time, primarily because of growing party-system fragmentation.

## 1. Introduction

Politics in Western democracies is increasingly shaped by distrust and hostility across party lines. This affective polarization [1] may prompt reluctance to have partisan opponents as neighbours, co-workers, or family members [2], discrimination against partisan opponents in economic transactions [3], and even the willingness to undermine democratic processes and condone violence in pursuit of political objectives [4].

Affective polarization is usually measured by comparing citizens’ (typically positive) feelings towards their own party versus their (frequently negative) feelings towards political opponents [e.g., 2, 5, 6], without accounting for the probability that partisans encounter co-partisans versus out-partisans. While such ‘feelings-based’ measures seem sensible for explaining some political phenomena, other social and economic consequences often linked to affective polarization–as well as political trust and satisfaction with democracy and media–may also depend on how frequently partisans interact with opponents relative to their in-group. Intuitively, the measure that best captures the social impacts of affective polarization can be stated as follows: If we randomly draw two individuals from a society, how warm is their interaction on average?

Of course, people do not interact at random. As individuals seek out others who share their views and values, real interaction partners will hold similar political views more often than is predicted by random chance [7, 8]. That is, societies will be somewhat segregated along party lines. However, self-segregating will be easier for members of large political parties [9]; the simple stochastic interaction probability does not translate into interactions one-to-one, but there is evidence that it does matter in practice [10, 11]. This is the case for intermarriage and partners’ matching on traits including occupation, race, or gender role attitudes [e.g., 12, 13], and also for politics and party preference. In Germany and the United States, cross-party couples are more common in regions and at times with greater political heterogeneity, i.e., higher stochastic cross-party interaction probabilities [14, 15]. At the party-level, as stochastic interactions would predict, supporters from smaller parties more often engage with out-partisans than supporters of large parties [16].

The above logic applies not only to personal interactions but also to participation in and exposure to political and public discourse. Like personal interactions, public discourse can be segregated to some degree, e.g., with people consuming media that mainly mirrors their own views. However, even in polarized settings, partisans from rival parties often consume the same national media [17–19]. Assuming these media are roughly representative of the country’s political landscape, smaller parties’ supporters are therefore more likely to confront media-based content they dislike.

According to this ‘probability-of-interaction-based’ perspective, supporters of large parties may enjoy more frequent harmonious social and economic interactions with their numerous co-partisans (individual-level), and feel represented by and be satisfied with the public media discourse (country-level). Smaller parties’ supporters, by contrast, may find their social and economic opportunities to be more restricted. Furthermore, unless small parties’ partisans confine themselves to politically segregated areas [20] and social circles, they are bound to interact frequently with out-partisans, where they may experience resentment and discrimination. In this regard, supporters of smaller, populist radical right parties have been found to be poorly socially integrated, highly affectively polarized, and to perceive that they experience discrimination from the media and the wider society [21–23]. We label our proposed measure, which accounts for both partisans’ feelings towards in-partisans and out-partisans and the probability of exposure to each partisan group, a measure of *affective fractionalization*.

By integrating fractionalization into the affective polarization literature, we extend previous research including the study of ethnic and cultural composition of a society. Here, higher fractionalization levels are associated with greater risks of conflict and lower societal cohesion [24, 25]. The widely-used index of ethnolinguistic fractionalization captures the probability that two randomly chosen individuals belong to different groups [12, 25, 26]. This measure has been extended to include the average ethnic or cultural similarity between two randomly drawn individuals in a society, which has been used as a proxy for the easiness of interaction between them [e.g. 26, 27]. Our proposed affective fractionalization measure accounts for both the probability of interaction and a direct measure of feelings, because some of the negative consequences linked to affective polarization in politics–notably those relating to partisans’ social and economic interactions, and possibly to democratic satisfaction and trust in government–are plausibly driven by both partisans’ feelings towards partisan in-groups and out-groups, and by how likely they are to interact with the members of each group.

We first review existing fractionalization measures and discuss the types of outcomes that may be most closely linked to our affective fractionalization measure. We then apply our measure to survey data from the Comparative Study of Electoral Systems from 20 Western publics over the period 1996–2019, and compare it to the affective polarization measures used in previous studies. These comparisons suggest four conclusions.

First, publics in less fractured party systems–notably the United States, which features two dominant parties–tend to display lower affective fractionalization levels than the publics in more fractured party systems. In the case of the US, partisans’ relative hostility towards out-partisans is somewhat mitigated by the fact that they are exposed to fewer opponents and more co-partisans, compared to the partisans of the smaller parties found in more fractionalized party systems. We find that in cross-national perspective the US scores as less affectively fractionalized than most other Western publics.

Second, because our affective fractionalization measure incorporates partisans’ feelings, the publics where partisans feel less hostile towards out-partisans also tend to display lower affective fractionalization levels, independently of party system fractionalization. Notably, the Dutch public, in which partisans express comparatively warm evaluations of opponents, scores as one of the least affectively fractionalized publics in our study–even though the Dutch party system is highly fractionalized [28].

Third, we find that affective fractionalization has intensified over time. This is consistent with concerns over the increasingly contentious nature of democratic politics across Western polities. Our measure allows us to decompose this increase into its constituent parts–rising negative affect towards out-parties and increased party system fragmentation. We find that in most countries, growing party system fragmentation drives over-time increases in affective fragmentation more than increasingly negative affect does: i.e., growing affective fractionalization is driven primarily by changes in the expected frequencies of cross-party interactions, not by changes in the warmth of these interactions.

Finally, in making comparisons within rather than across mass Western publics, we find that the supporters of smaller parties, who are likely to interact disproportionately often with out-partisans, can expect to encounter a more hostile environment than larger parties’ supporters. This is especially true for the partisans of small, extremist parties–such as radical populist parties of the left and the right–who are often intensely disliked by mainstream partisans, and who can expect to interact frequently with these mainstream opponents.

## 2. Political affect, personal relationships, public discourse, and societal fractionalization

At the individual level, negative political affect may undermine personal interactions in the family, the workplace, or in social interactions such as dating [29–31]. In fact, scholars and commentators often worry about and analyze polarization *because* it has consequences for personal relationships that might eventually harm social cohesion more generally [14, 32–35]). Dislike of out-groups–whether defined in terms of politics, religion, region, ethnicity, or language–can damage social interactions, both by dissuading people from entering into meaningful interactions and by decreasing the quality of the interactions they do pursue.

While it is not surprising that political dissonance dissuades partisans from entering into social interactions in the politicized sphere of Twitter [36], this avoidant behavior is also found in apolitical contexts such as dating (Nicholson *et al*., 2016; Huber and Malhotra, 2017), roommate searches [37], and the labor market [30]. Political sorting and segregation are, however, far from absolute and many people have family members, friends, romantic partners, or economic transaction partners who support different parties. Negative party-political affect may damage these relationships. In the sphere of economic transactions and career decisions, people tend to charge higher prices to out-partisans [38] and are less likely to recommend them for a scholarship [39]. Moreover, qualitative studies show how serious political dissent affects friendships, family, and romantic relationships [40].

Even more than for direct personal interactions, political sorting in media consumption is far from absolute. Also in the digital age, people with different political preferences consume much of the same national, mainstream media [17, 18]. Fractionalization shapes the macro-level, public discourse and media, and in politically fractionalized societies people confront more speakers and media commentators associated with political opponents. In extreme cases this could drive some citizens away from mainstream media and towards highly biased sources, which could boost echo chambers that harm social cohesion [17, 41].

Existing affective polarization studies mostly capture the intensity of dislike toward political opponents, but not the relative size of the in-group versus the out-groups. Measures such as the system-level affective polarization deployed in Reiljan (2020) and Gidron, Adams, and Horne (2020), or Wagner’s (2021) Weighted Spread of Scores measure account for different out-parties’ relative sizes, but not for the size of the partisan’s in-party compared to its opponents (the out-parties). However, the probability of encountering in- versus out-partisans determines what proportion of potential romantic partners, business partners, friends, or roommates partisans potentially screen out. It also determines how many economic contacts will be compromised by political dislike, and how many couples and families might see their relationships undermined by political disagreements.

We do not propose that our affective fractionalization measure should replace commonly used affective polarization measures, which are defined exclusively in terms of the difference between affect towards one’s in-party versus its opponents. Some political phenomena, such as partisans’ willingness to bend the rules or to condone violence that advances their party’s objectives, may be best predicted by the intensity of out-party dislike alone. Moreover, a partisan’s tendency to discriminate against an out-partisan in economic or social interactions occurs plausibly depends on this inter-party hostility. What we argue is that it also matters how often these types of economic/social interactions occur, i.e., the relative probabilities with which partisans can expect to interact with opponents versus co-partisans. Our affective fractionalization measure incorporates these probabilities.

## 3. Affective polarization and affective fractionalization: Conceptualizing the different measures

How is affective fractionalization similar to, and how does it differ from the widely used affective polarization measure? First, both measures build upon the widespread thermometer rating scales that survey respondents use to rate their feelings towards different political parties, which correlate well with other measures of affect including social distance [42]. The feeling thermometer question asks respondents to rate a group on a “thermometer” scale, often from either 0–10 or 0–100 where higher ratings denote warmer feelings. Our data relies on the question as it appears in the Comparative Study of Electoral Systems: “I’d like to know what you think about each of our political parties. After I read the name of a political party, please rate it on a scale from 0 to 10, where 0 means you strongly dislike that party and 10 means that you strongly like that party.” In the formulas for both affective polarization and affective fractionalization, each respondent’s in-party and out-party thermometer ratings must be identified. One approach is to rely on CSES questions that ask respondents to directly identify their preferred party, if any. However, this approach excludes respondents who do not state a party affiliation. Therefore we follow Wagner [43] and define the “in-party” as the party for which respondents expressed the warmest thermometer rating (if the respondent had a tie between two parties, but listed one of them as their “closest” party when prompted, we assigned that party as their party ID. Finally, if they tied between multiple parties, and did not list a favorite from them, we randomly assigned one of their highest-ranking parties as their party ID). the online supplement reports results for the alternative approach using the CSES party identification questions.

In line with several previous affective polarization studies [e.g. 5, 6], we rely on a difference-based measure of political affect. The warmth of an interaction is thereby defined by the difference between the thermometer rating of people’s in-party versus their rating of the interaction partner’s party. This procedure assigns ingroup interactions the value zero and defines them as neutral. This makes sense given experimental research showing that the negative discrimination against the political out-group is much larger than the positive discrimination for the political in-group [30, 37, 44, 45]. Moreover, calculating the ingroup-out-group difference alleviates issues of differential item functioning, i.e., that some survey respondents generally tend towards higher or lower thermometer ratings than others, which has been shown to be a serious problem in analyses of survey respondents’ party thermometer ratings [46]. (The survey item asks respondents to rate political parties as opposed to rating the party’s supporters. However, the item seems to be a reliable proxy for respondents’ expressed feelings towards partisans. Harteveld (2021, p. 5) analyses this issue for the Netherlands, finding that "the average dislike towards parties correlates well with average dislike towards their respective partisans: the correlation is r = .95.”Tichelbaecker et al. (forthcoming) also reports significant correlations between respondents’ thermometer ratings of parties and of their preferred social distance from these parties’ supporters, across ten party systems.)

We then calculate the country- and party-level affective polarization and fractionalization scores. While there is not one textbook solution to calculating affective polarization, much research uses formulas that are identical or very similar to the one printed in Reiljan (2019). For comparison, here are the formulas to calculate affective polarization, as used in Reiljan (2019), and affective fractionalization, side by side:

**Table:** 

	*Affective Polarization (APol)*	*Affective Fractionalization (AFrac)*
*Party-level*	$APol_{A}=\sum_{i=B}^{K}\left(\;affect_{AA}-affect_{Ai}\right)*\frac{\;vote\;share_{i}}{1-vote\;share_{A}}$	$AFrac_{A}=\sum_{i=A}^{K}\left(\;affect_{AA}-affect_{Ai}\right)*\;vote\;share_{i}$
*Country-level*	$APol=\sum_{i=A}^{K}APol_{i}*vote\;share_{i}$	$AFrac=\sum_{i=A}^{K}\;AFrac_{i}*\;vote\;share_{i}$

Where A to K are the parties in an election and affect_Ai_ denotes the affect of supporters of party A towards party i. Higher values on the affective polarization (*APol*) or the affective fractionalization (*AFrac*) index signify colder, i.e., more hostile interactions.

The central difference in the formulas is what the affect gap (difference between the rating of the in-party vs. an out-party: *affect*_*AA*_—*affect*_*Ai*_) is multiplied with. *APol* multiplies the affect gap with the out-party’s vote share relative to the total vote share of all parties minus one’s own party ($\frac{vote\;share_{i}}{1-vote\;share_{A}}$). Intuitively, *APol* accounts for the share of random interactions that are with party_*i*_’s supporters as a proportion of all out-partisan interactions. By contrast *AFrac* multiplies *affect*_*AA*_—*affect*_*Ai*_ with the raw out-party’s vote share (*vote share*_*i*_), thereby accounting for the share of random interactions that are with party_*i*_’s supporters as a proportion of all interactions including those with co-partisans. This difference in calculation implies a fundamentally different logic. *AFrac* is the average party-based affect in interactions between any two randomly chosen partisans, including pairs of co-partisans, while *APol* is the average party-based affect for the subset of partisans’ random interactions with out-partisans only. *APol* increases with the degree of hostility between supporters of different parties, but is independent of the probability of interacting with co-partisans. The affective fractionalization measure increases with both the degree of hostility between supporters of different parties and with the probability of interactions with out-partisans. Indeed, *AFrac* mathematically equals the multiplication of *APol* with the probability that any random encounter is cross-partisan.

**Fig 1:**
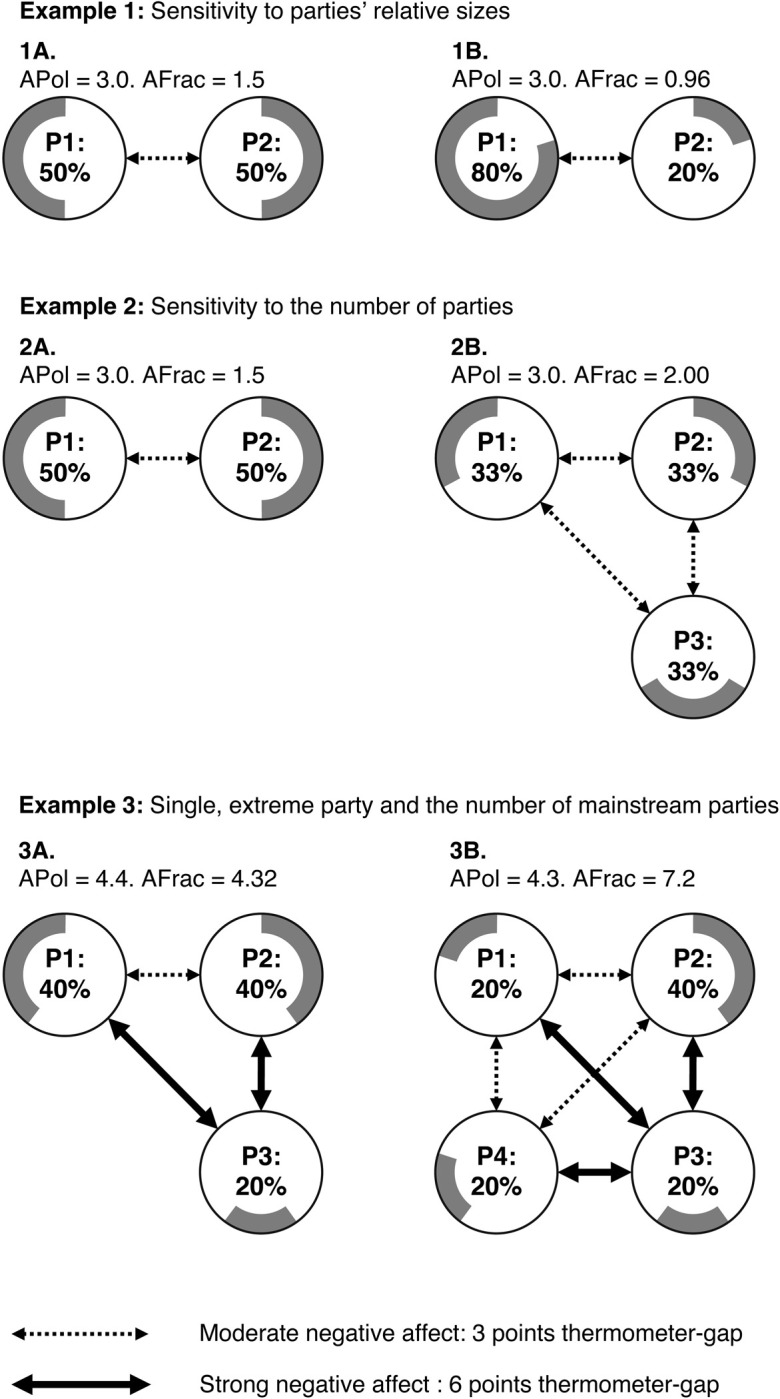
Illustrating empirical circumstances under which affective polarization and affective fractionalization diverge.

To illustrate these differences, Fig 1 shows three sets of comparisons between hypothetical party systems. As outlined below, in the first two cases *APol* assigns equal values, i.e., equal degrees of affective polarization, to both party systems, while the *AFrac* values change sharply. In the third case, *APol* and *AFrac* even point in opposite directions.

### Example 1: Sensitivity to parties’ relative sizes

Example 1 compares two different two-party systems featuring parties P1 and P2, in which partisans display moderate levels of hostility towards out-parties, i.e., the difference between partisans’ in-party and out-party thermometer ratings is three points on the 0–10 thermometer scale. However, in the two-party system displayed in Example 1A the parties are equal-sized, while in Example 1B Party P1 is dominant, with 80% of the country’s partisans. In both scenarios, the standard *APol* measure will return the same score of 3.0 for the intensity of affective polarization. However, the *AFrac* measure returns different scores for these two scenarios: In Example 1A, partisans have an 0.5 probability of encountering out-partisans in random interactions (in such interactions there is a moderately negative affect, i.e., a 3-point thermometer-gap) and an 0.5 probability of encountering co-partisans (where the affect is neutral, i.e., a 0 points thermometer gap). Hence the intensity of affective fractionalization (*AFrac*) is 1.5 (*Afrac* for Party 1: 3*0.5 = 1.5; 50% cross-party interactions with moderate negative affect [3] and 50% in-party interactions with neutral affect [0]). The intensity of affective polarization is 3.0, because the thermometer-gap is 3 in all cross-partisan interactions, and co-partisan interactions are not considered (*Apol* for Party $1:3*\frac{0.5}{1-0.5}=3*1=3$; all of the cross-partisan interactions have moderate negative affect [3]).

In Example 1B on the other hand, where Party P1 is dominant, partisans’ probabilities of encountering out-partisans in random interactions is only 0.32, and the *AFrac* measure drops to 0.96, i.e., the intensity of affective fractionalization is much lower in Example 1B than in Example 1A. However the affective polarization value (*APol*) is the same in Examples 1B and 1A.

While no major Western democracy has such a dominant party as in Example 1B, these comparisons are more relevant for issue-based polarization, where two camps oppose each other [28, 35]. There are many contemporary examples of policy debates or social movements that have divided Western polities, including the Yellow Vest movement in France, the Catalonian Separatist movement in Spain, the UK’s Brexit debate, and the protests in Ottawa, Canada over the government’s coronavirus policies. Some of these have divided electorates roughly evenly; others have featured strong majorities on one side, a difference that the affective fractionalization measure considers.

For instance, imagine that the 2016 Referendum on leaving the European Union had not only taken place in the United Kingdom, but also in Spain which has many fewer EU sceptics. Imagine further that Remainers and Leavers had disliked each other equally in both countries. The ratio of the two camps in the United Kingdom roughly corresponds to the 50:50 scenario of Example 1A while in Spain, it corresponds to the 80:20 scenario of Example 1B [47]. Therefore, an EU-referendum in Spain would likely not spark the same unrest, protest, and divisions as in the United Kingdom. The affective fractionalization measure, which accounts for the likelihoods of partisans’ interactions with co-partisans (or for pro-leavers interactions with other pro-leavers, etc.), distinguishes between scenarios where the sides are roughly evenly divided versus those where one side enjoys a strong majority. The affective polarization measure does not.

The first example also illustrates how the differences between *APol* and *AFrac* matter at the party or group level. In a system with a single dominant party (or when there is a large majority on one side of a bitter policy debate), those on the majority side are likely to enjoy much more frequent encounters with co-partisans in economic and social settings and to find the distribution of viewpoints presented in the mainstream media much more hospitable than those on the minority side. The *AFrac* measure accounts for this difference at the level of different groups or partisan constituencies, by incorporating the relative proportions of expected co-partisan interactions.

### Example 2: Sensitivity to the number of parties

Consider Example 2 where we again assume that all partisans display moderate hostility towards out-parties, with the difference between partisans’ in-party and out-party thermometer ratings set at three thermometer points. However, Example 2A pictures a system of two equal-sized parties (as in Example 1A above), while Example 2B displays a system of three equal-sized parties P1, P2, and P3. In both scenarios, the standard *APol* measure returns the same score of 3.0 for the intensity of affective polarization. However, the *AFrac* measure returns a higher (i.e., more intensely fractionalized) score in the three-party scenario pictured in Example 2B, a value of *AFrac* = 2.0, compared to the two-party scenario in Example 2A for which *AFrac* = 1.5. This is because in the scenario in Example 2B, the three equal-sized parties are all smaller than the two equal-sized parties pictured in Example 2A, so that every partisan in Example 2B has a lower probability of encountering co-partisans than do the partisans in Example 2A. The partisans in the three-party system in Example 2B might therefore experience more frequent economic and social discrimination, have a smaller pool for dating and for roommates, and encounter fewer congenial viewpoints in mainstream media reports, compared to the partisans in the two-party system in Example 2A.

The comparison between the scenarios displayed in Examples 2A and 2B roughly captures the difference between the American two-party system and the contemporary Austrian party system, in which the three largest parties (the SPÖ, ÖVP, and FPÖ) have all received comparable levels of support in some recent elections while far out-distancing their smaller rivals. This measure also captures changes in party systems over time. Many European party systems have fractionalized in recent years, with declining support for mainstream parties while newer challenger parties increase in support.

### Example 3: The effect of a single, extreme party depends on the number of mainstream parties

The party landscape in many Western democracies includes several mainstream parties and a smaller number of populist and/or extremist parties. A typical pattern is that mainstream parties’ supporters express moderately negative to neutral affect towards each other, but strongly negative affect towards the populist radical parties. Examples 3A and 3B show such societies. In both cases, Party P3 is in extreme conflict with all the other parties: P3’s partisans assign out-party thermometer ratings that are six thermometer points lower than their in-party ratings, and reciprocally, mainstream partisans rate the out-party P3 six points lower than their in-party. Meanwhile, we set the difference between mainstream partisans’ in-party and out-party thermometer ratings of other mainstream out-parties at three thermometer points, as in our earlier examples. The only difference between Examples 3A and 3B is that in 3A, the mainstream party P1 has 40% of the partisans in the electorate, whereas in 3B this party has split into two parties, P1 and P4, with 20% each. Example 3B is *less* affectively polarized but *more* affectively fractionalized than 3A.

Here is an intuitive account for these differences, explained from the perspective of a supporter of party P1. Imagine a talk show with five politicians as guests who each represent 20% of the population. In 3A, a supporter of party P1 likes two of the guests, moderately dislikes two guests, and despises one guest. In 3B, that person would like only one guest, moderately dislike three guests, and also despise one guest. The number of guests they despise is equal in both societies, but the number of guests they moderately dislike is larger in 3B. This means that the average affect towards the out-group partisans is more moderate in 3B, i.e., less hostile. Due to this, society 3B is actually *less* affectively polarized. However, the average affect towards all of the five guests is more negative in society 3B. The value for affective fractionalization accounts for this and is higher for 3B.

## 4. Data

We first calculate country-level measures of affective fractionalization using the Comparative Study of Electoral Systems (CSES) data. For our sample, we include all Western democracies that appear with at least two elections in the CSES. We exclude Belgium because there, respondents are only asked to rank parties within their own ethnic communities. We include information on 92 election studies from 20 countries between 1996–2019. Table 1 displays the countries and election-year surveys in our study, which includes every CSES election survey which has been released to date across the 20 Western democracies in our study.

**Table 1:** Countries and elections included in the analyses.

Country	Elections included
Australia	1996, 2004, 2007, 2013, 2019
Austria	2008, 2013, 2017
Canada	1997, 2004, 2008, 2011, 2019
Denmark	1998, 2001, 2007
Finland	2003, 2007, 2011, 2015, 2019
France	2002, 2007, 2012, 2017
Germany	1998, 2002, 2005, 2009, 2013, 2017
Great Britain	1997, 2005, 2015, 2017
Greece	2009, 2012, 2015
Iceland	1999, 2003, 2007, 2009, 2013, 2016, 2017
Ireland	2002, 2007, 2011, 2016
Israel	1996, 2003, 2006, 2013
Netherlands	1998, 2002, 2006, 2010
New Zealand	1996, 2002, 2008, 2011, 2014, 2017
Norway	1997, 2001, 2005, 2009, 2013, 2017
Portugal	2002, 2005, 2009, 2015, 2019
Spain	1996, 2000, 2004, 2008
Sweden	1998, 2002, 2006, 2014, 2018
Switzerland	1999, 2003, 2007, 2011
United States	1996, 2004, 2008, 2012, 2016

## 5. Results

### 5.1. The two elements of affective fractionalization: Out-party dislike and probability of out-party interactions

**Fig 2:**
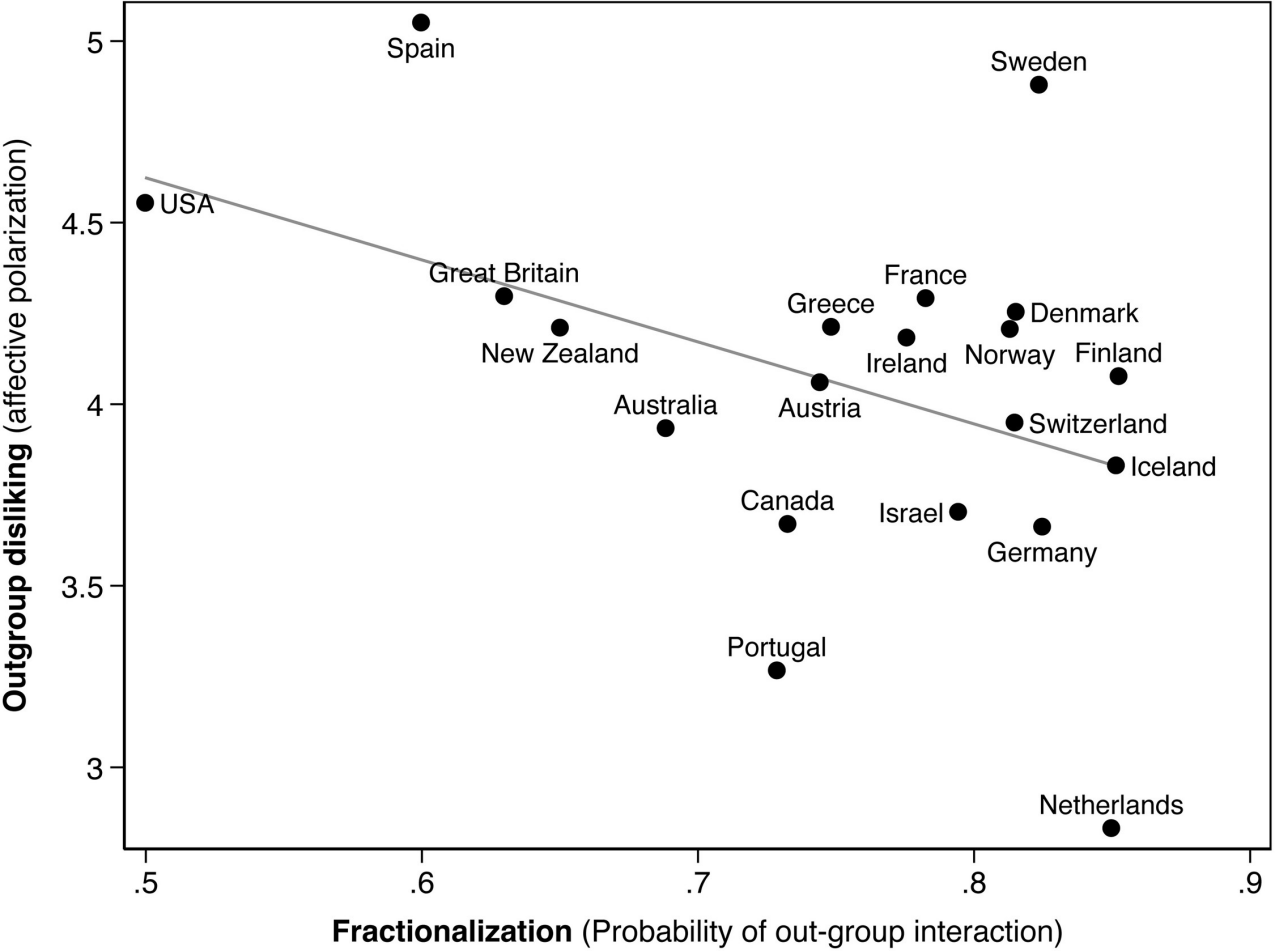
The two components of affective fractionalization: the random likelihood of interacting with out-partisans (the horizontal axis) plotted against out-group dislike (the vertical axis). Data are for the most recent CSES election survey (see Table 1 above).

Conceptually, our affective fractionalization measure comprises two components. The first is the degree to which a country’s party system is fractionalized, defined as the likelihood that partisans interact with out-partisans as a proportion of all random partisan interactions. The second, affective polarization, is the difference in affect between co-partisan interactions and out-partisan interactions, defined as the difference in mean thermometer scores respondents assigned to out-parties versus their in-party. Fig 2 shows these components, displaying the odds of a random interaction with an out-partisan (as a proportion of all-partisan interactions) on the X-axis, and the average difference between in-partisan and out-partisan affect in such an interaction on the Y-axis. There, higher values represent a greater affective difference, i.e., more negative feelings towards out-parties compared to the in-party. The plotted data utilizes the most recent CSES country-year for each country in our study, given in Table 1 above.

The countries differ considerably in their probabilities of out-partisan interactions. In the United States, the random probability that a partisan interacts with an out-partisan is roughly 0.5. However, the two-party American system is an outlier among the Western polities in our study: in every other polity, the likelihood of out-partisan interactions exceeds 0.6, and in several countries–including the Netherlands, Germany, and the Scandinavian countries–the proportion of random interactions with out-partisans exceeds 0.8. There is a visible divide between clusters of countries. The Anglo-American countries tend to display lower likelihoods of out-partisan interactions compared to the other polities in our study, plausibly because all of them (except New Zealand) feature single-member district (SMD) voting systems that tend to depress the number of viable political parties [48].

The degree of difference in affect between co-partisan interactions and out-group interactions also displays sharp cross-national variation, but without clear clusters by region or electoral system. Consistent with past research [5, 49], Spanish and American citizens tend to express particularly intense hostility towards out-parties, relative to in-parties (e.g., Iyengar et al. 2019), while Dutch partisans express by far the warmest out-party evaluations. Fig 2 displays a negative correlation (r = -.43, p = .06) between the likelihood of interactions with out-partisans and hostility towards out-parties, i.e., cross-partisan encounters tend to be somewhat less hostile, on average, in countries where partisans’ likelihoods of interacting with out-partisans are higher. This pattern is consistent with other research finding that more proportional electoral systems tend to display less intense out-party animosity [50].

### 5.2. Level of affective fractionalization by country

**Fig 3:**
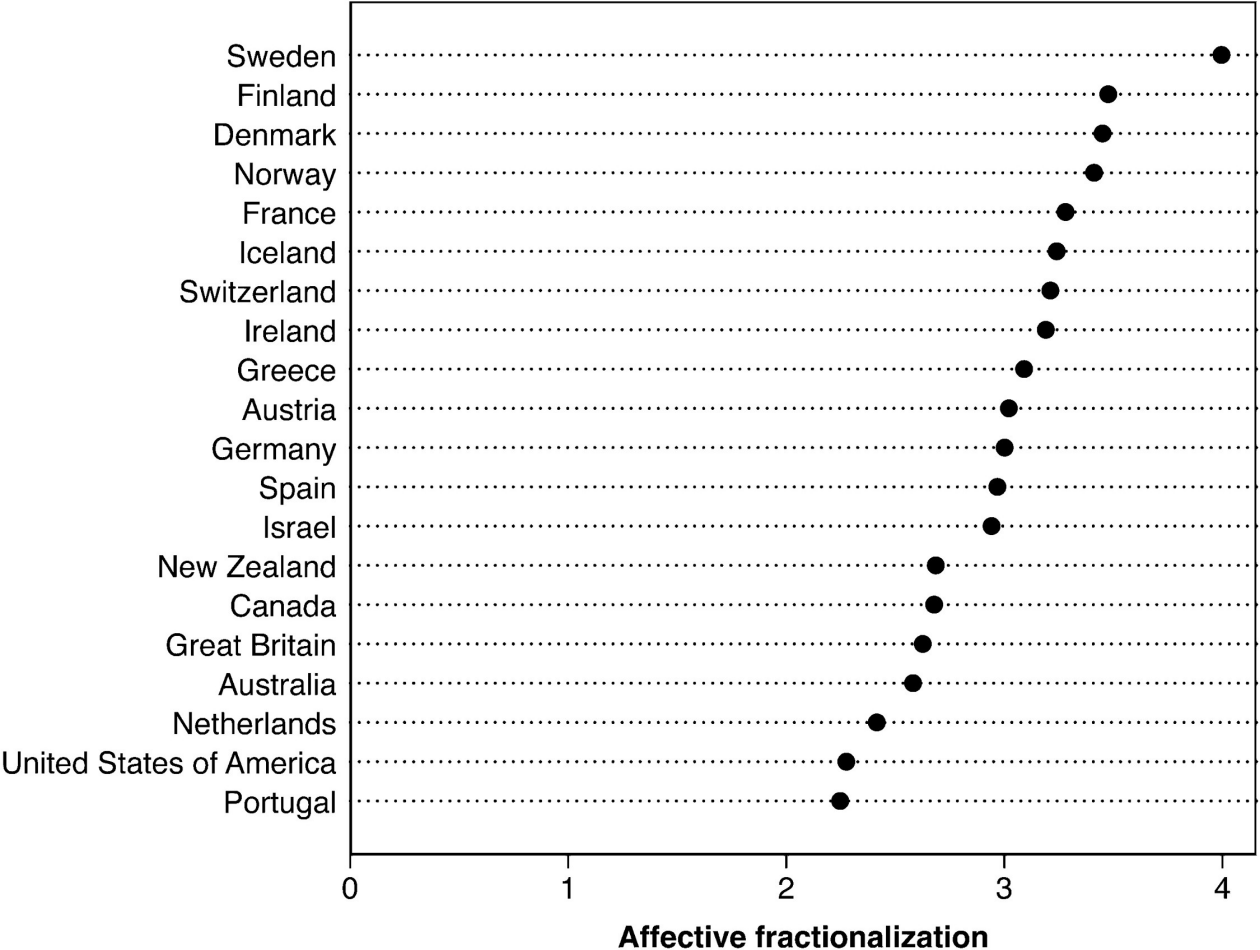
Levels of affective fractionalization by country. Higher values denote more negative affect. Data are for the most recent Comparative Study of Electoral System election survey (see Table 1 above).

Fig 3 combines the two components plotted in Fig 2 above, average out-party dislike and the random probability of interacting with an out-partisan, to present our measure of affective fractionalization (*AFrac*), again using the most recent CSES election survey year in each country. As a reminder, this measure can be interpreted as follows: If we randomly draw two individuals from a society, how warm is their interaction, on average? We see that according to our *AFrac* measure, the three least affectively fractionalized publics are those of Portugal, the United States, and the Netherlands–though for different reasons. The United States scores low on affective fractionalization because even though Americans tend to report comparatively intense animosity towards political opponents, i.e., they are affectively polarized, they are less likely to interact with these opponents than are the citizens in any other Western public. The Netherlands also scores low on the *AFrac* measure, but for the opposite reason from the United States: even though Dutch partisans are very likely to interact with political opponents because their party system is highly fractionalized, they express far less hostility towards out-parties, on average, than do the citizens in any other public in our study. Portugal, which also displays low affective fractionalization, features a situation that is intermediate from The Netherlands and the US: Portugal’s party system is much more fractionalized than the US but less so than The Netherlands, while Portugal’s citizens express far less hostility towards out-parties than do American citizens, but more out-party hostility than the Dutch (Portuguese respondents’ expressed out-party dislike was far less intense in the 2019 CSES election study than in the earlier waves of the survey). The low affective fractionalization scores for the American, Dutch, and Portuguese publics illustrate that our measure is sensitive to both the levels of hostility that partisans express towards opponents and to the probability with which partisans can expect to interact with opponents. Note, moreover, that the Finnish, Icelandic, and Swiss polities display comparatively high affective fractionalization levels due to their large party systems, even though their partisans do not express especially intense hostility towards out-parties.

### 5.3. Comparing country levels of affective fractionalization vs. affective polarization

**Fig 4:**
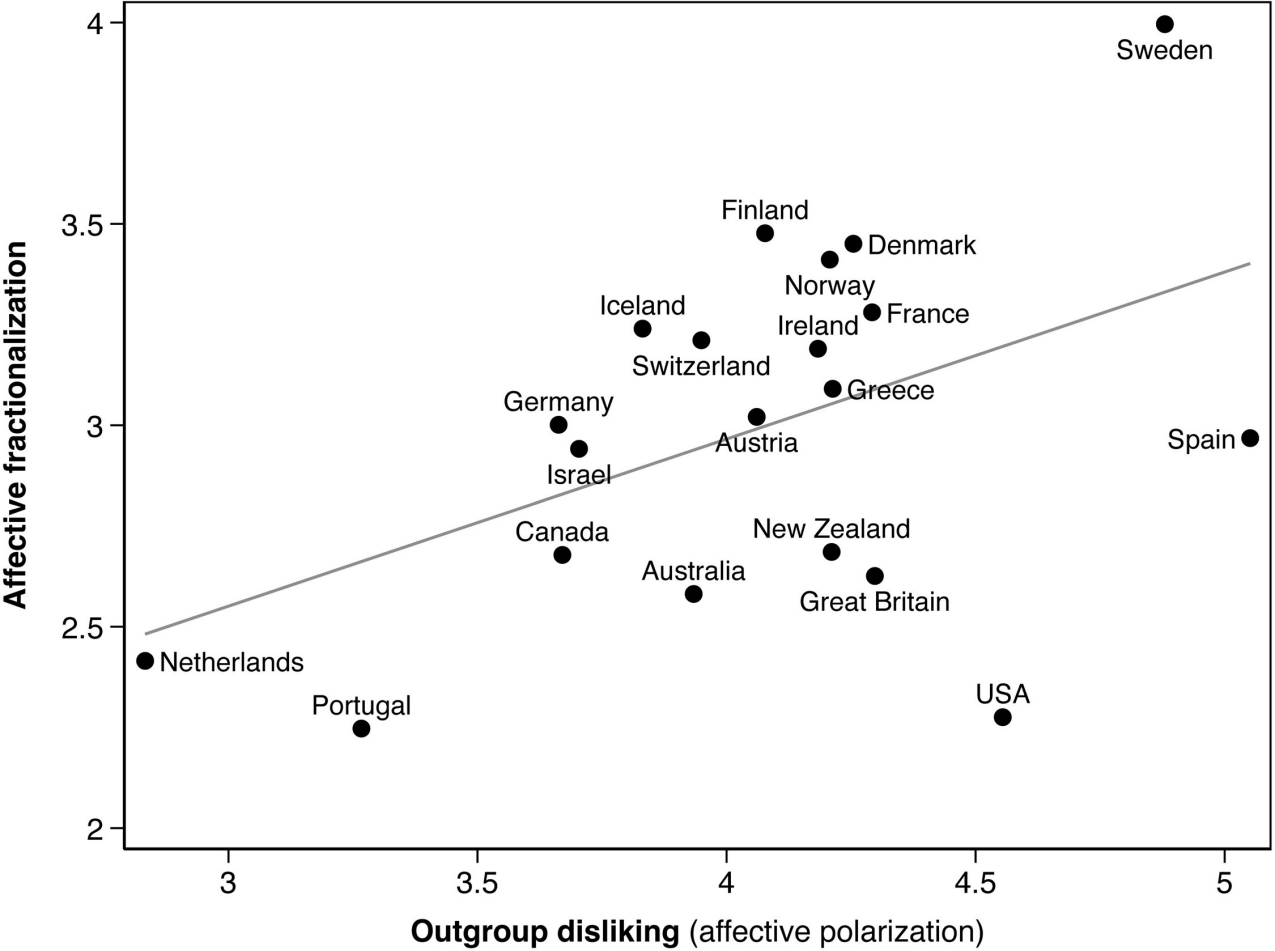
The levels of affective fractionalization plotted against levels of out-group dislike (affective polarization) by country. Data are for the most recent CSES election survey (see Table 1 above).

Fig 4 plots countries’ affective polarization levels (*APol*, the horizontal axis) versus their affective fractionalization levels (*AFrac*, the vertical axis), where higher values along each axis denote more intense polarization/fractionalization. The figure displays the most recent CSES year for each country, reported in Table 1 above. The fitted line displays, as expected, a positive association between the observed country levels of *APol* and *AFrac* (*r* = .47, p < .05), i.e. the countries where partisans express more intense out-party hostility (relative to their in-party evaluations) also tend to display more intense affective fractionalization. Yet the country-level relationship between *APol* and *AFrac* is only moderately strong because the *AFrac* measure also accounts for the expected probability of interactions with out-partisans (relative to in-partisans). Thus, societies that are affectively polarized need not be affectively fractionalized, with the US as the most prominent example.

### 5.4. Time-trends in affective fractionalization and its components

**Fig 5:**
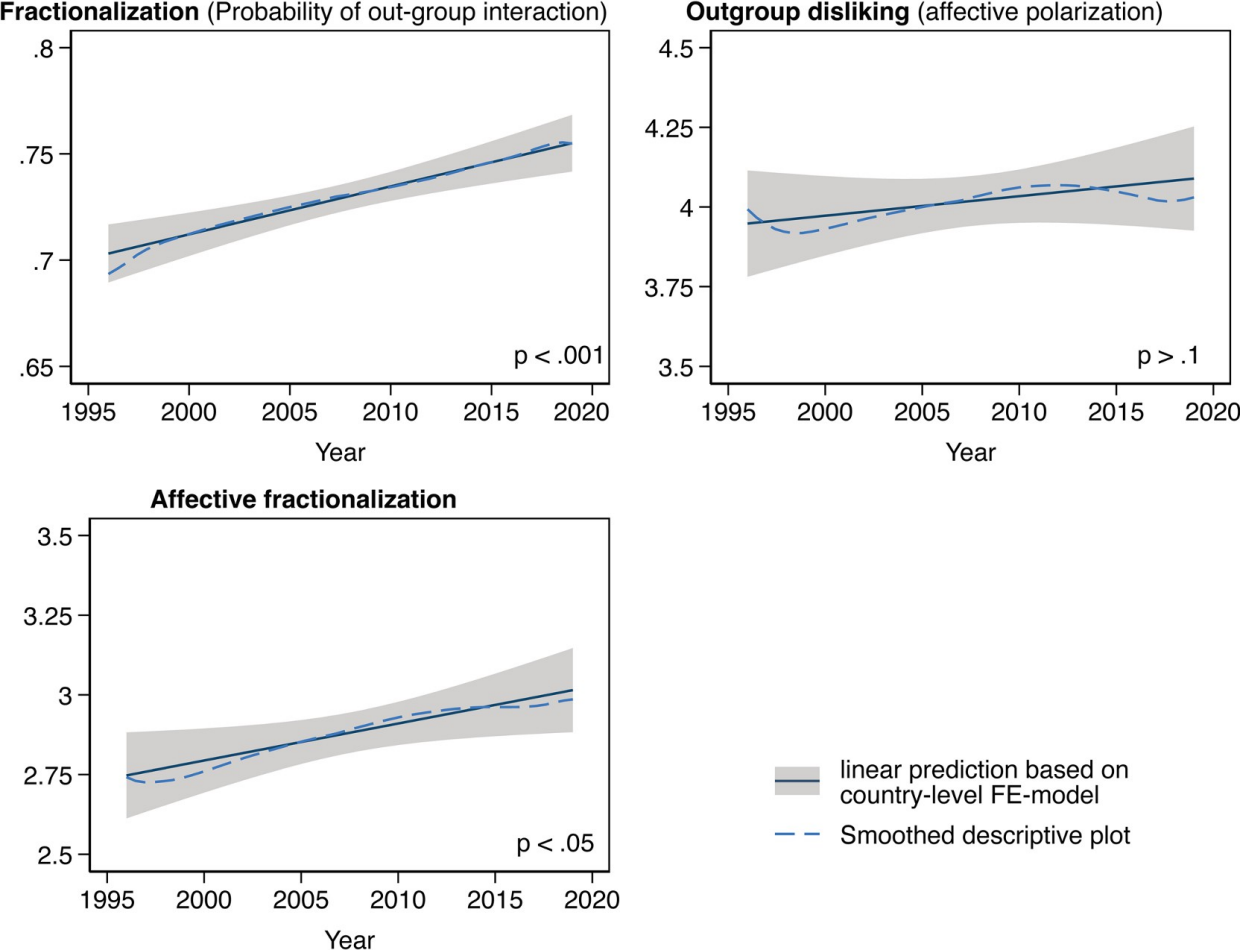
Time trends in affective fractionalization (the lower panel) and its two components, the random likelihood of interacting with out-partisans (the upper left panel) and out-party dislike (the upper right panel). The smoothed plot is generated via local linear smoothing. These computations are over all the CSES country-election surveys listed in Table 1 above.

Fig 5 displays time trends in affective fractionalization and its two components, party system fractionalization and out-party dislike, computed on all the CSES country-election surveys listed in Table 1 above. The solid lines with 95% confidence intervals are from linear regression models with fixed-effects at the country level. Therefore, these trends are only estimated based on within-country changes. Over time, the increase in party system fractionalization (displayed in the upper left-hand panel) is substantial and roughly linear. Across all countries combined, the predicted probability of a cross-party interaction rises from 70.8% in 1996 to 75.1% in 2019. The difference between in- and out-group party ratings (the out-group dislike measure, displayed in the upper right-hand panel) does not show a clear time trend. The solid regression line indicates a slight increase which is not statistically significant. Together, these factors add up to a significant increase in affective fractionalization–which is the product of the affective polarization measure and the random probability of interacting with an out-partisan–from 2.7 to 3.0 between 1996 and 2019 (the lower left-hand panel), primarily due to the rising probabilities of citizens interacting with partisan opponents.

Fig A6 in S1 File presents a country-by-country overview of changes in affective fractionalization (*AFrac*) and its components. Affective fractionalization has increased in 14 out of 20 countries. In some countries including the United States, Finland, the United Kingdom, and Switzerland, *AFrac* is rising because cross-party interactions are becoming colder, while in many other countries–including Australia, Denmark, France, Germany, and Greece–*AFrac* has intensified primarily due to increasing party system fragmentation which increases the likelihood of interactions with opponents.

### 5.5. Affective fractionalization and its components at the party-family level

Our measure can be used to analyze affective fractionalization at the level of individual parties or party families. Table 2, row one, displays the probability that someone supporting a given party encounters someone who supports the same party in a random interaction, for citizens subdivided according to the party family to which their preferred party belongs, computed for the most recent CSES election-year survey for each country in our study. Overall, supporters of the typically large conservative and social democratic parties have relatively high probabilities of meeting co-partisans in random interactions (0.28 and 0.24, respectively), whereas this likelihood is below 0.10 for supporters of the (typically) smaller green and radical left parties. The next row displays the intensity of negative affect that partisans can expect to receive from people who prefer a different party (“Negative partisan affect received in out-group interactions only”), i.e., affective polarization, where higher numbers denote more intense dislike. We see that radical left and especially radical right parties’ supporters receive the most intense dislike from rival parties’ supporters, on average (see also [28, 51, 52], while conservative parties’ supporters also receive comparatively intense out-partisan dislike. The last row displays the intensity of negative affect that people can expect to receive in random interactions with all citizens including co-partisans, i.e., affective fractionalization. Here we see that conservative parties’ supporters receive comparatively mild hostility levels in random interactions, because they are the most likely to interact with co-partisans which mitigates their opponents’ hostility. By contrast radical left and right parties’ supporters are highly unlikely to randomly interact with co-partisans, which combined with their opponents’ hostility generates intensely negative expected affect in random interactions, compared to other parties’ supporters. This is consistent with previous research findings that radical right partisans tend to experience alienation and feelings of rejection from society at large (Gidron and Hall, 2020).

**Table 2:** Components of affective fractionalization by party family.

	Radical Left	Green	Social Democrat	Liberal	Christian Democrat	Conser-vative	Radical Right
Vote share (random probability of ingroup interaction)	0.09	0.07	0.24	0.13	0.13	0.28	0.13
*Negative partisan affect received in out-group interactions only*	4.66	3.65	3.61	3.49	3.68	4.20	5.14
*Negative partisan affect received in all interactions*	4.28	3.40	2.69	3.03	3.19	3.03	4.46

Notes: Higher values denote more *negative* affect. These computations are for the most recent CSES election survey for each country in our study (see Table 1 above). The computations in columns 2–3 assume that citizens randomly interact with political opponents’ supporters (row 2) and with all citizens including co-partisans (row 3).

## 6. Discussion

We have developed a new measure, affective fractionalization, which may better capture some of the negative social dynamics ascribed to affective polarization. We do not propose this concept as a *replacement* for the traditional measures of affective polarization, but instead as a *complement*. For measures of cross-party hostility in the public, the affective polarization measure seems appropriate. However, for personal interactions and societal cohesion–among the main reasons why scholars and the public worry about polarization–the affective fractionalization-perspective is important.

A cohesive society is characterized by trust, tolerance, and cooperation between its members [27, 53]. Negative partisan political sentiments reduce trust between different parties’ supporters [44] and might lead people to avoid interacting and cooperating [30, 31]. The extent to which political dislike affects cohesion depends not only on the intensity of the dislike or discrimination that party supporters direct towards opponents, but also on how likely people are to encounter members of political out-groups. Building on the literature on ethnic fractionalization, we argue that it matters how often political dislike causes people to avoid contact, distrust others, or discriminate against them.

Our measure also allows us to decompose affective fractionalization into two components, party system fragmentation and affective polarization. We find that both components have intensified over the last 25 years across western publics, with the overall rise in affective fractionalization primarily driven by the growing fragmentation of party systems, particularly outside of the United States which remains the only strict two-party system among western democracies. In this regard, our measure casts American society in a more positive light than does the standard affective polarization measure, for while Americans express comparatively intense hostility towards opponents, they are less likely to interact with these opponents than are the citizens in other western publics.

Fractionalization and the intensity of political dislike could be interrelated. Following the contact hypothesis, fractionalization might reduce out-party animosity through increased contact probabilities [54]. Indeed, cross-party relationships are more common in contexts with higher fractionalization [14, 15], and such relationships may qualify for the type of context where contact reduces animosity and increases mutual understanding. However, to our knowledge, the effects of close relationships on party-political dislike remain untested. In line with the idea that fractionalization might decrease out-party dislike, our findings showed a negative correlation between the two.

Of course, the calculated probabilities we applied to compute our fractionalization index do not necessarily equal people’s actual frequency of meeting members of the political out-group, as people often select into social interactions with in-group members. We observed the highest degree of fractionalization in Finland, but it could be that some Finns live in rather segregated neighbourhoods where they primarily meet co-partisans (individual-level). However, when they consume national media (country-level) they also confront public discourse determined by the degree of fractionalization in the country as a whole. That is, even people whose neighbourhoods and everyday interactions are segregated [20] are still affected by and “feel” the situation at the country level.

Moreover, the level of fractionalization affects how easy or costly it is for people to segregate. In low-fractionalization societies, such as the United States, it is easier to self-select into surroundings where one’s own party is dominant. This is harder in politically fractionalized societies such as Finland or Germany, particularly for smaller parties’ supporters. For our index of affective fractionalization to be a useful analytical tool, it is not necessary that the computational probabilities equal the actual interaction frequencies, only that these probability be sufficiently correlated with the frequencies of actual encounters in everyday life and/or the exposure in media and public discourse. This correlation should hold between and within countries. Thus we expect (1) the average Finn or German to have higher exposure to out-partisans than the average American [as found by 16], and (2) the average Finn or German today to have greater exposure to out-partisans than they had several decades ago–arguments that are consistent with the recent literature on cross-partisan interactions [8, 15].

The more (politically) segregated a society is, the lower the local level of affective fractionalization will tend to be, all else equal. Lower local affective fractionalization levels are thereby not an unalloyed good, particularly if they are “achieved” through segregation that may be socially undesirable (or even immoral). Finally, segregation affects the relevance of spatial scale for measurement. With high segregation, fractionalization can be high at the national level but low at the sub-national level. A measurement at the sub-national level would then more faithfully capture these interpersonal relations.

Future research may analyze which outcomes are better explained by affective fractionalization versus the standard affective polarization measure. We have speculated that the polarization concept may prove superior for explaining partisans’ willingness to bend democratic rules or condone violence against opponents. However, affective fractionalization may prove as the more powerful concept for explaining outcomes such as strain on personal relationships and economic transactions, satisfaction with political discourse and democracy, and social cohesion.

To date, the affective polarization literature has emphasized the question of how much party supporters dislike the political out-group members they interact with in social and economic situations. We extend this perspective to emphasize how often people can expect to interact with political opponents, relative to how often they interact with co-partisan allies. We believe that both the intensity of cross-party hostility and the probabilities of outs-partisan interactions matter when assessing the consequences of politically-motivated dislike causes across Western publics. Compared to affective polarization, our affective fractionalization construct may provide a more complete measure of how personal interactions and societal cohesion are affected by party-political animosity.

## Supporting information

S1 File (PDF)
