# Supplementary material for: How warm are political interactions? A new measure of affective fractionalization
Source: PLoS One. 2024 May 14;19(5):e0294401. doi: 10.1371/journal.pone.0294401 (PMC11093341; doi:10.1371/journal.pone.0294401)
Supplement: S1 File — (PDF) [file pone.0294401.s001.pdf]

APPENDIX TO:

How Warm Are Political Interactions? A New Measure of Affective Fractionalization

Ansgar Hudde[1]✉, Will Horne[2], James Adams[3] & Noam Gidron[4]

[1] University of Cologne, Institute of Sociology and Social Psychology

[2] Georgia State University

[3] University of California, Davis

[4] The Hebrew University of Jerusalem

✉ **hudde@wiso.uni-koeln.de**

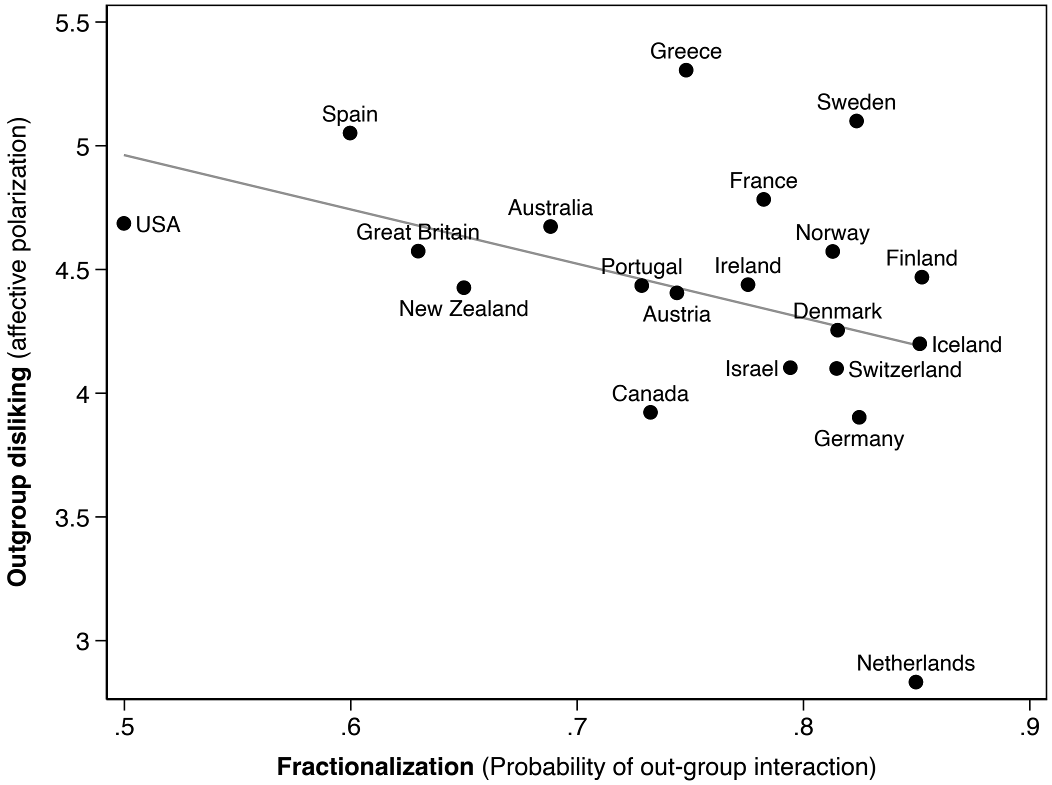

Figure A1: Alternative modeling strategy: "in-party" defined through partisanship (instead of as the best-rated party). The two components of affective fractionalization: the random likelihood share of out-group interactions and out-group disliking. Data are for the last observation per country (year 2015, on average).

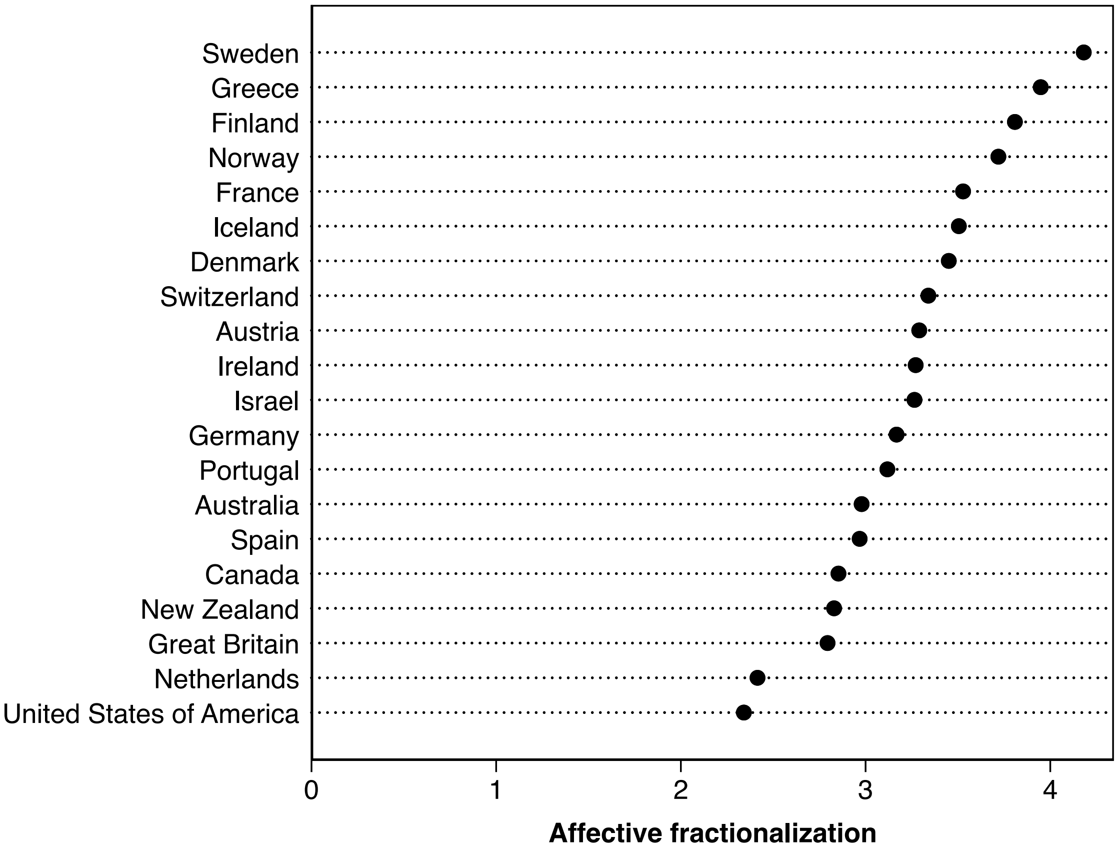

Figure A2: Alternative modeling strategy: "in-party" defined through partisanship (instead of as the best-rated party). Levels of affective fractionalization by country. Higher values indicate more negative affect. Data are for the last observation per country (year 2015, on average).

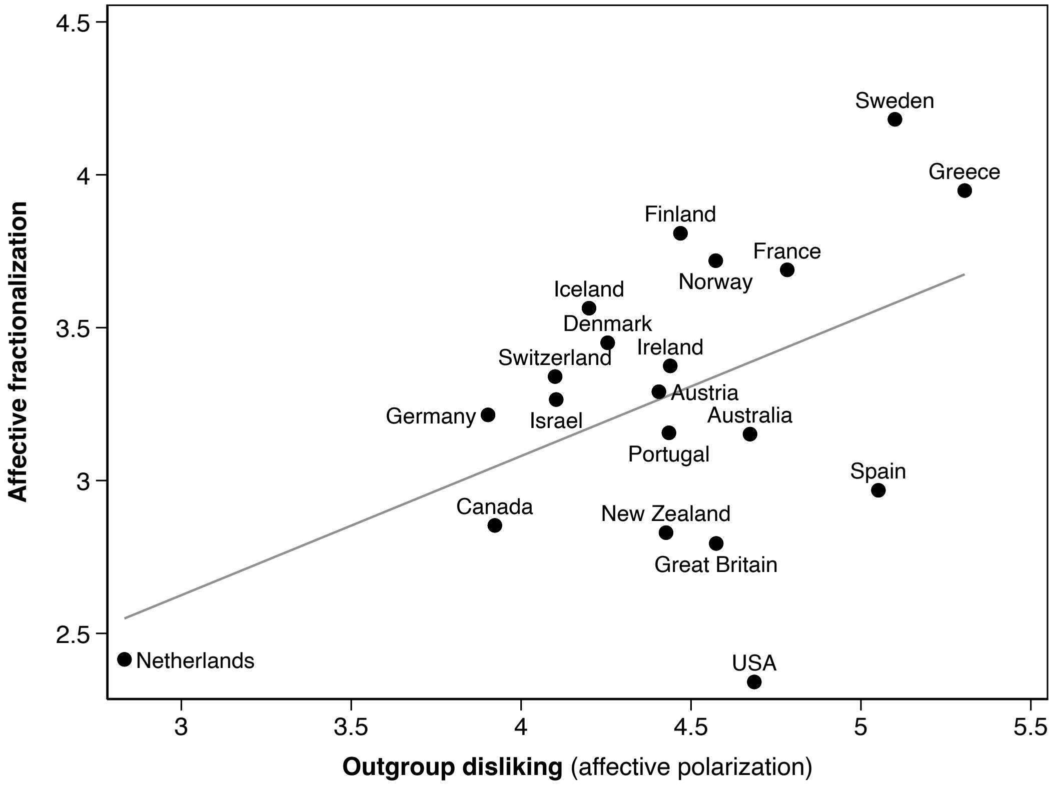

Figure A3: Alternative modeling strategy: “in-party” defined through partisanship (instead of as the best-rated party). The levels of affective fractionalization and out-group disliking (affective polarization) by country. Data are for the last observation per country (year 2015, on average).

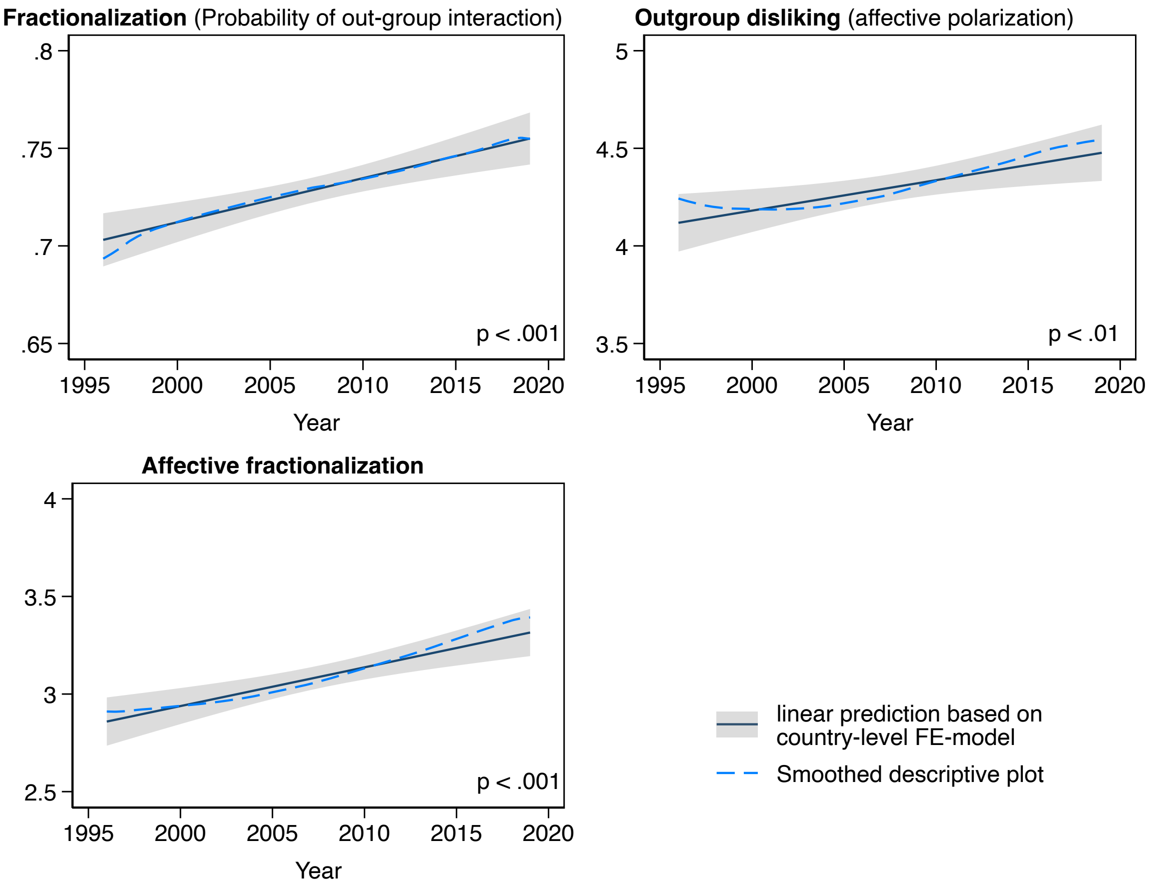

Figure A4: Alternative modeling strategy: "in-party" defined through partisanship (instead of as the best-rated party). Time trends in affective fractionalization and its two components. The smoothed plot is generated via local linear smoothing.

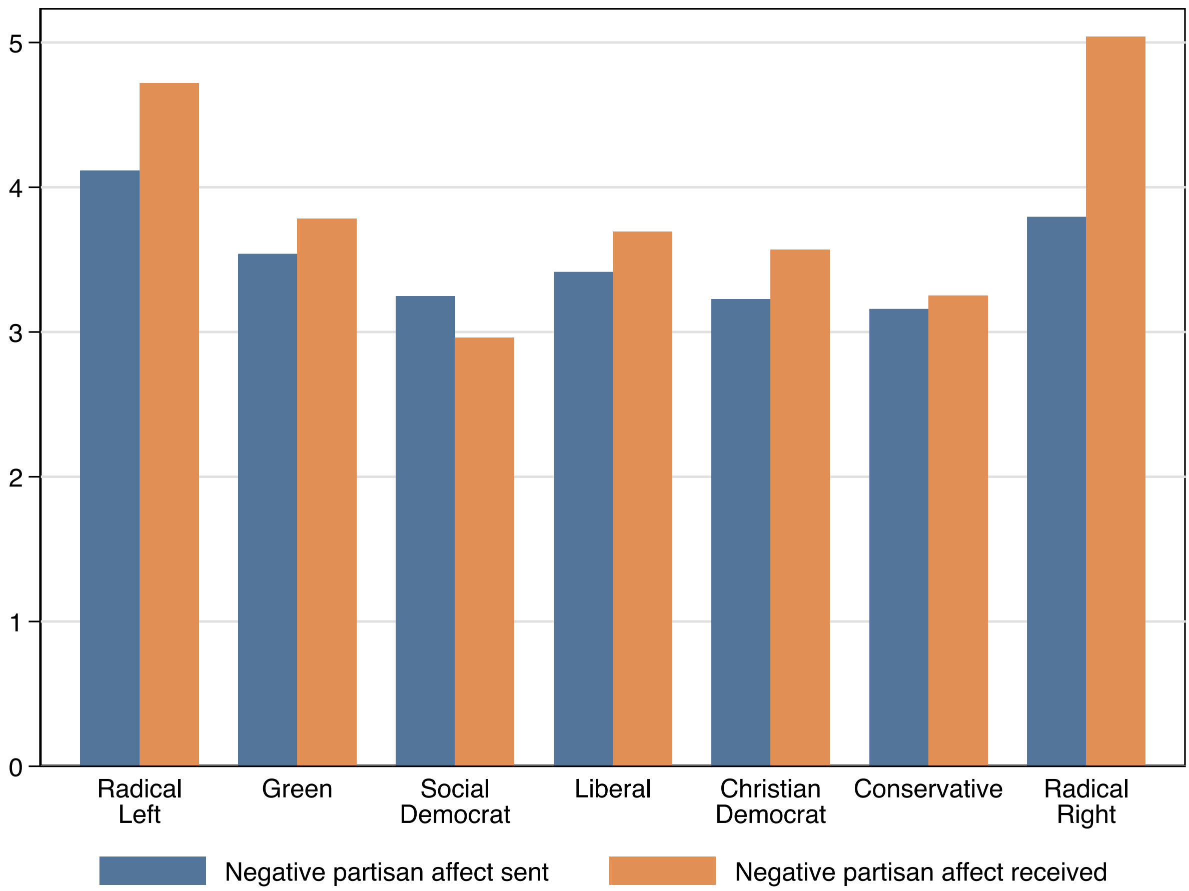

Figure A5: Alternative modeling strategy: "in-party" defined through partisanship (instead of as the best-rated party). Negative partisan affect sent and received in interactions with any partisan (in- or out-partisan), by party family.

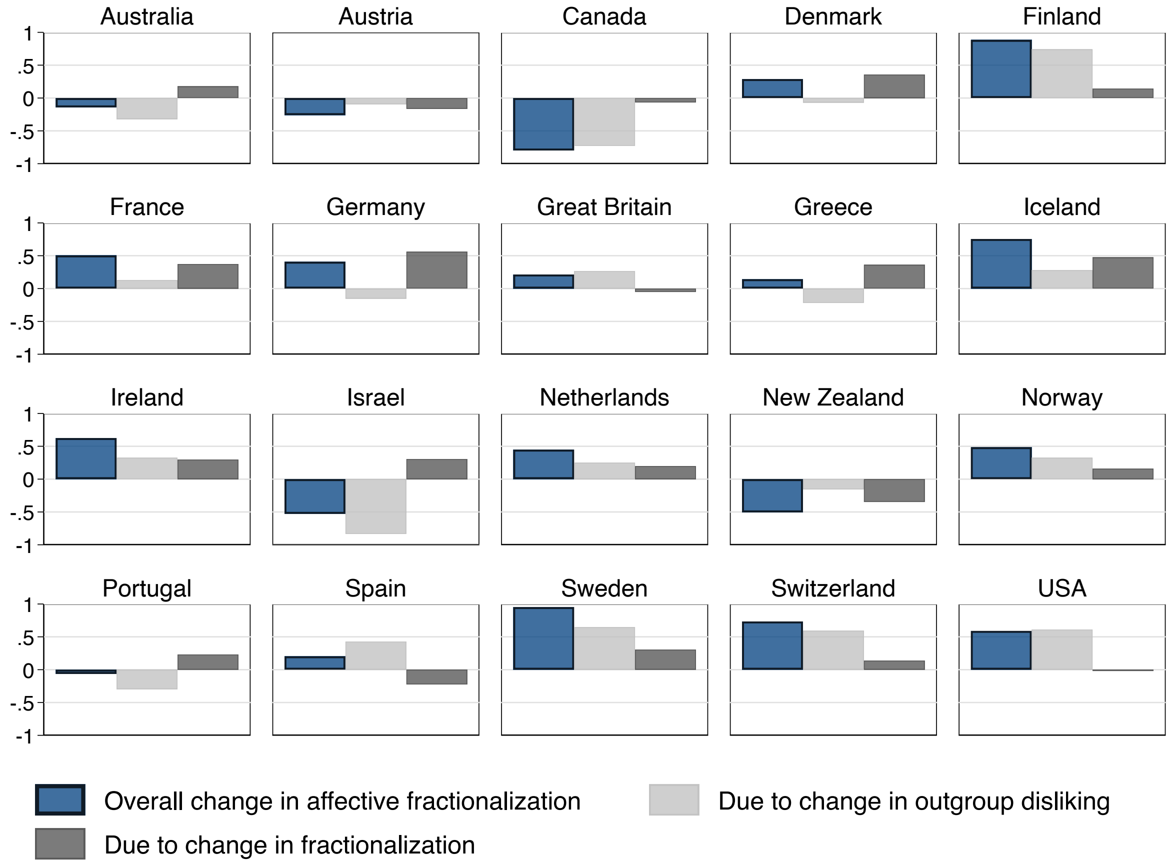

Figure A6: Time trends in affective fractionalization by country, decomposed to changes in party system fractionalization and changes in out-group affect.

Figure A6 shows the over-time change in affective fractionalization for each country, and displays how much of this change is driven by changes in party system fractionalization versus changes in out-group affect. In each case, the country's first and last CSES election survey is compared. In 14 out of 20 countries, affective fractionalization has increased. In some cases – including the United States, Finland, Norway, the United Kingdom, and Switzerland – this increase is primarily because cross-party interactions are becoming colder, while in other cases – including Australia, Denmark, France, Germany, Iceland, Greece, Israel, and Portugal – affective fractionalization has intensified primarily due to growing party system fragmentation.
